# CD38 mediates nicotinamide mononucleotide base exchange to yield nicotinic acid mononucleotide

**DOI:** 10.1016/j.jbc.2025.108248

**Published:** 2025-01-31

**Authors:** Romanthi Madawala, Jasmine L. Banks, Sarah E. Hancock, Lake-Ee Quek, Nigel Turner, Lindsay E. Wu

**Affiliations:** 1 School of Biomedical Sciences, UNSW Sydney, Kensington, NSW, Australia; 2 Victor Chang Cardiac Research Institute, Darlinghurst, NSW, Australia; 3 School of Clinical Medicine, UNSW Sydney, Kensington, NSW, Australia; 4 School of Mathematics and Statistics, The University of Sydney, Sydney, NSW, Australia

**Keywords:** CD38, nicotinamide mononucleotide (NMN), nicotinamide adenine dinucleotide (NAD+) biosynthesis, stable isotopes, metabolomics, nicotinamide adenine dinucleotide (NAD+), nicotinic acid mononucleotide (NaMN), base exchange, transglycosidase, transglycosylase, transglycosylation, Preiss–Handler pathway, salvage pathway

## Abstract

Nicotinamide mononucleotide (NMN) is a widely investigated metabolic precursor to the prominent enzyme cofactor NAD^+^, where it is assumed that delivery of this compound results in its direct incorporation into NAD^+^*via* the canonical salvage/recycling pathway. Surprisingly, treatment with this salvage pathway intermediate leads to increases in nicotinic acid mononucleotide (NaMN) and nicotinic acid adenine dinucleotide, two members of the Preiss–Handler/*de novo* pathways. In mammals, these pathways are not known to intersect prior to the production of NAD^+^. Here, we show that the cell surface enzyme CD38 can mediate a base-exchange reaction on NMN, whereby the nicotinamide ring is exchanged with a free nicotinic acid to yield the Preiss–Handler/*de novo* pathway intermediate NaMN, with *in vivo* small molecule inhibition of CD38 abolishing the NMN-induced increase in NaMN and nicotinic acid adenine dinucleotide. Together, these data demonstrate a new mechanism by which the salvage pathway and Preiss–Handler/*de novo* pathways can exchange intermediates in mammalian NAD^+^ biosynthesis.

NAD^+^ is a prominent oxidation-reduction enzyme cofactor that declines during ageing and physiological challenges such as DNA repair, senescence, and inflammation (1, 2, 3, 4, 5, 6). Over the past decade, there has been strong interest in restoring NAD^+^ levels as a therapeutic approach to overcoming age-related diseases including frailty, infertility, and impaired muscle function (7, 8, 9, 10, 11). These approaches have included the use of the metabolic precursors nicotinamide riboside (NR) (12, 13, 14) and nicotinamide mononucleotide (NMN) (15), both of which are amidated precursors that act as substrates for NAD^+^ biosynthesis *via* the salvage or recycling pathways—in the case of NR, first entering the pathway following its phosphorylation *via* the NRK pathway (16, 17, 18). The other well-characterized pathways to NAD^+^ biosynthesis in mammals are the Preiss–Handler pathway, which utilizes nicotinic acid as a substrate for the production of nicotinic acid mononucleotide (NaMN) (19, 20), and the *de novo* pathway, which converts the amino acid tryptophan into NaMN, both of which are converted into nicotinic acid adenine dinucleotide (NaAD) prior to the final step of its conversion to NAD^+^.

One core chemical difference between these pathways is that the salvage pathway utilises amidated intermediates, whereby the nicotinamide ring contains an amine group as a sidechain from the pyridine ring (Fig. 1). In contrast, the Preiss–Handler/*de novo* pathways utilize intermediates containing nicotinic acid, which contains a carboxylic acid sidechain, and could be considered as deamidated counterparts to amidated precursors in the salvage pathway. In mammals, these pathways do not intersect, and there are no mammalian enzymes known to mediate the deamidation of salvage pathway intermediates into their deamidated counterparts (Fig. 1*A*). Despite this, administration with the amidated intermediates NR, NMN, and their reduced equivalents NRH and NMNH, leads to striking increases in the deamidated metabolites NaMN and NaAD (21, 22, 23, 24, 25, 26, 27, 28). The question of how treatment with the amidated intermediate NMN leads to an increase in levels of the deamidated intermediates NaMN and NaAD (23, 24) is the core goal of this investigation, as it could point to overlap in these pathways, and provide an update to these textbook pathways of NAD^+^ biosynthesis.

**Figure 1:**
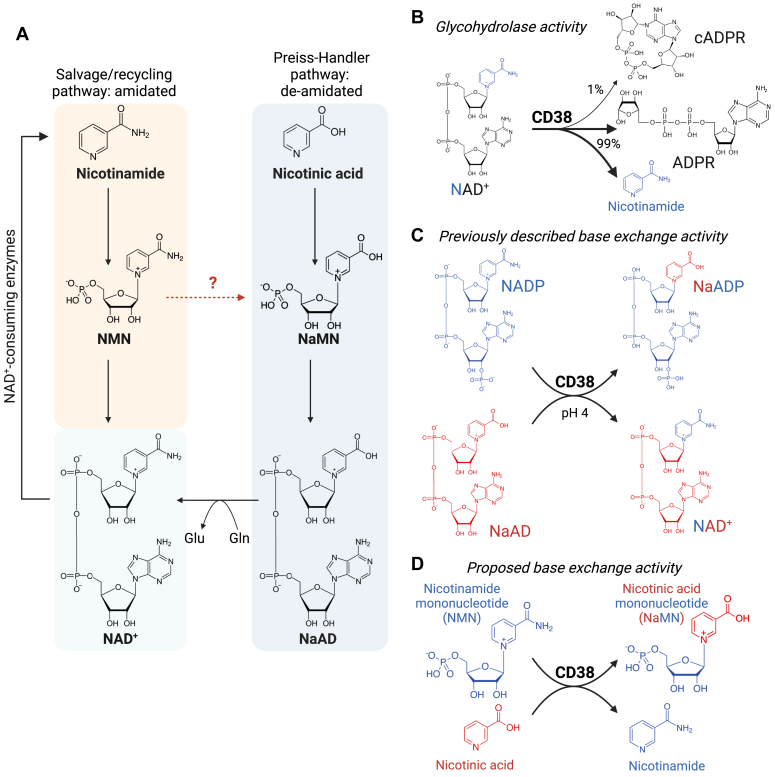
**Mammalian NAD**^**+**^ **biosynthesis and proposed role for CD38**. *A*, NAD^+^ can be synthesized *via* the salvage pathway, which recycles the amidated precursor nicotinamide *via* an amidated intermediate, nicotinamide mononucleotide (NMN). In the Preiss–Handler pathway, nicotinic acid is incorporated into NAD^+^*via* the deamidated intermediates nicotinic acid mononucleotide (NaMN) and nicotinic acid adenine dinucleotide (NaAD). Treatment with the amidated precursor NMN can increase levels of the deamidated intermediates NaMN and NaAD. *B*, the enzyme CD38 is well-studied for its NAD^+^ glycohydrolase activity, cleaving NAD^+^ into free nicotinamide and ADP ribose (ADPR). *C*, CD38 also has base-exchange activity toward NADP^+^, yielding the Ca^2+^ signaling intermediate NaADP^+^. *D*, this investigation identified NMN and nicotinic acid as new base-exchange substrates for CD38, yielding NaMN and explaining the crossover between the amidated salvage/recycling pathways and the deamidated Preiss–Handler/*de novo* pathways of NAD^+^ biosynthesis.

One mechanism for the link between these amidated and deamidated pathways of NAD^+^ biosynthesis is a role for the gut microbiome in mediating the deamidation of these precursors when they are delivered orally (24, 29, 30), due to the expression of bacterial deamidase enzymes that are lacking in mammals, such as the enzyme PncC, which deamidates NMN into NaMN (31). Recently, we used a stable isotope tracing strategy to show that orally delivered NMN could be incorporated into NAD^+^ *via* the deamidated *de novo* pathway and that incorporation *via* this pathway was reduced following ablation of the gut microbiome through antibiotics treatment (24). While these data showed a reduction in NMN incorporation *via* the NAD synthase (NADS)-dependent deamidated pathway during antibiotics treatment, this did not lead to a complete ablation of incorporation *via* this route. Further, early results from the field suggested the existence of mammalian enzymes that could convert NMN into NaMN (32, 33). Another complication is that previous results from ourselves and others using stable isotope-labeled precursors have identified only a small fraction of double-labeled NR or NMN being incorporated into NAD^+^ intact (24, 29, 34, 35), with the majority of NAD^+^ labeling only containing a single label from the ribose or nicotinamide groups, suggesting that these precursors could undergo gastrointestinal metabolism into free nicotinamide prior to their delivery and incorporation into NAD^+^ in other tissues.

One additional explanation for these observations could be that in addition to microbial deamidation (24, 29, 30), the amidated and deamidated pathways of NAD^+^ biosynthesis could intersect *via* base exchange (transglycosylation), rather than deamidase activity, whereby the entire nicotinamide ring would be exchanged for a nicotinic acid. This was recently described for the membrane-bound enzyme BST1/CD173, which could convert NR into nicotinic acid riboside (NaR) *via* exchange of the entire nicotinamide ring for a nicotinic acid (36). This has also been described for the NAD^+^ glycohydrolase SARM1, which can mediate base exchange on NAD^+^ with nicotinic acid and other similar compounds (37). The cell surface enzyme CD38 is well studied for its role as an NAD^+^ glycohydrolase (Fig. 1*B*) in inflammation, infertility, senescence, and ageing (1, 2, 3, 38, 39, 40), and there is strong interest in the use of small molecule CD38 inhibitors as a strategy to preserve NAD^+^ levels (2, 39, 40). In addition to its glycohydrolase activity, CD38 also has base-exchange activity (Fig. 1*C*), whereby it uses NaAD as a nicotinic acid donor for base exchange with NADP^+^ to yield the Ca^2+^ mobilizing signaling intermediate, nicotinic acid adenine dinucleotide phosphate (NaADP) in endolysosomes (41, 42, 43, 44, 45, 46, 47, 48), and is required for NaADP production in mammalian tissues (44). CD38 also has prominent affinity toward NMN, acting as a glycohydrolase for its breakdown into free nicotinamide *in vitro* and *in vivo* (2, 49), and is capable of carrying out base-exchange reactions on NMN for drug-like heterocyclic substrates (50).

Given both its affinity for NMN (2, 49), and its base-exchange activity toward other NAD^+^ metabolites (41, 42, 44, 45, 48, 50), we hypothesized CD38 could mediate base-exchange activity toward NMN (Fig. 1*D*), helping to resolve the question of how the oral administration of the amidated precursor NMN can lead to sharp increases in the deamidated precursors NaMN and NaAD (23, 24). Here, we demonstrate that small molecule inhibition of CD38 abolishes the spike in NaMN and NaAD caused by NMN treatment, and further show that this is due to direct base-exchange activity between NMN and free nicotinic acid, with similar activity toward a series of other nicotinic acid analogs. These findings demonstrate an important link between the amidated salvage pathway and the deamidated Preiss–Handler/*de novo* pathways for NAD^+^ synthesis in mammals.

## Results

In the current investigation, we sought to identify additional mechanisms through which exogenous treatment with the amidated NAD^+^ precursor NMN could elevate NaMN and NaAD levels (23, 24), which also occurs during treatment with NR (21, 25). Our previous investigations (24) focused on the gain or loss of isotope labels on the amide group of NAD^+^ in animals treated with or without antibiotics, where we had sought to identify whether there was deamidation of this group by nonmammalian enzymes expressed by the gut microbiome. Here, we sought to investigate whether the formation of NaMN and NaAD following NMN treatment could also occur independently of direct deamidation. Instead, we deduced that if NMN could undergo endogenous base exchange with a free nicotinic acid, this mechanism could also explain the formation of NaMN and NaAD.

**Figure 2:**
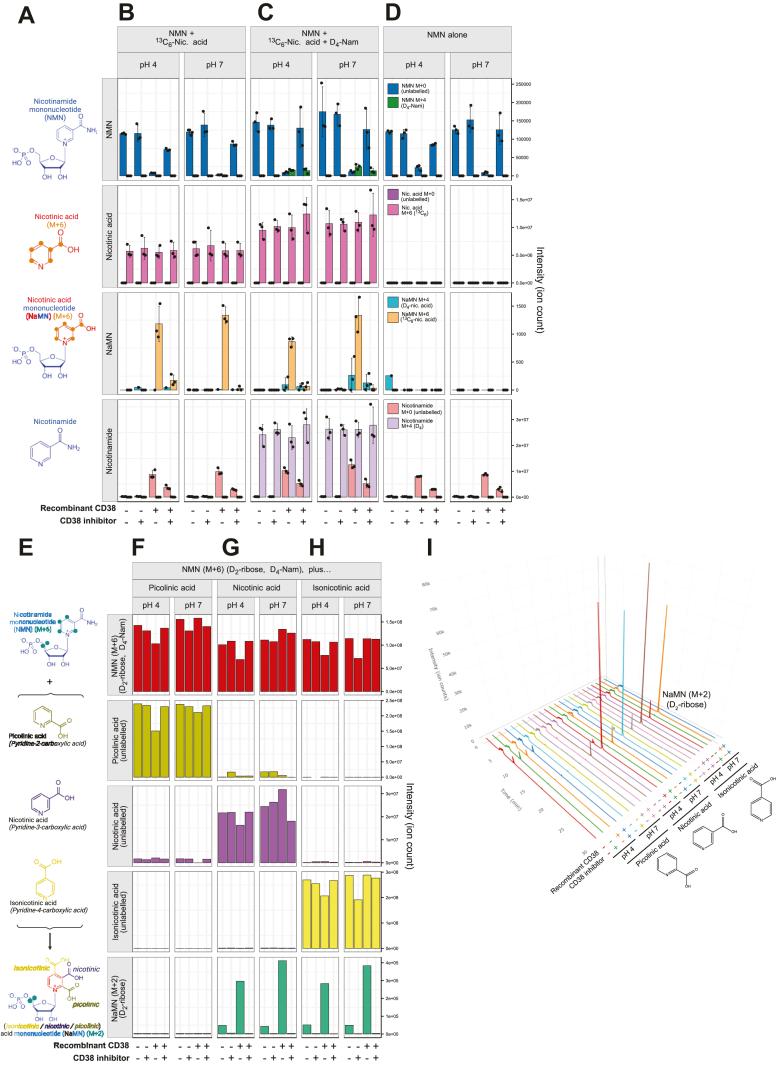
**CD38 mediates NMN base exchange with nicotinic acid**. *A*, experimental scheme to identify NMN base exchange. Unlabeled NMN (M + 0) and ^13^C_6_-labeled–nicotinic acid (M + 6) are coincubated, and the formation of M + 6–labeled nicotinic acid mononucleotide (NaMN) is indicative of base exchange of the unlabeled nicotinamide group from NMN for an M + 6–labeled nicotinic acid. *B*, targeted mass spectrometry for these metabolites in the presence or absence of recombinant CD38 protein, with or without cotreatment with the small molecule CD38 inhibitor **78c**, showing CD38-dependent formation of M + 6–labeled NaMN. This base exchange is preferential for nicotinic acid over nicotinamide, as (*C*) coincubation of unlabeled NMN with both ^13^C_6_-nicotinic acid (M + 6) and D_4_-nicotinamide (M + 4) leads to the greater formation of M + 6 NaMN over M + 4 NMN. *D*, technical control to show the absence of labelled NaMN in the absence of ^13^C_6_-nicotinic acid (M + 6). *E*, scheme to test whether CD38-mediated base exchange is selective to nicotinic acid. Double-labeled NMN containing D_2_-ribose and D_4_-nicotinamide, for an overall mass shift of M + 6 was coincubated with nicotinic acid or its structural orthologs picolinic acid or isonicotinic acid, which are identical in molecular weight. CD38-mediated base exchange would result in exchange of the D_4_-nicotinamide label for an unlabeled group, resulting in the formation of a product with a molecular weight identical to NaMN plus an M + 2 mass shift, due to retention of the ribose label. Base exchange was not observed for (*F*) picolinic acid, but was observed for (*G*) nicotinic acid and (*H*) isonicotinic acid. *I*, chromatograms showing the detection of M + 2 ribose-labeled NaMN in each group. Data points represent technical replicates within a single experiment, and experiments were performed three times. Error bars are SDs. NMN, nicotinamide mononucleotide.

We had hypothesized that if base exchange on NMN could occur, that one candidate would be the membrane-bound enzyme CD38, which has been the subject of intense study in the field of NAD^+^ biology due to its role as an NAD^+^ glycohydrolase. This enzyme also has base-exchange activity toward NADP^+^ (41, 42, 45, 48) to mediate the formation of NaADP, and can utilize drug-like cyclic substrates for the base exchange of NMN (50). We first investigated this using recombinant human CD38 enzyme (UniProt P28907, Val54–Ile300), to test whether it could mediate the conversion of amidated NAD^+^ precursors to their deamidated counterparts, and *vice versa*. We used targeted mass spectrometry to measure the production of reaction products, including the putative base-exchange product NaMN. One challenge to this approach is that NMN and NaMN are very close in molecular weight, and to overcome the issue of potential misidentification due to similar molecular weights, we utilized stable isotope-labeled substrates containing ^13^C_6_ (M + 6) or D_4_ tetra-deuterated (M + 4) labeling of the nicotinyl ring (Fig. 2*A*), allowing greater mass separation for the clearer identification of newly formed base-exchange products. If base exchange were to occur, incubation of NMN with isotope-labeled nicotinic acid should result in the formation of labeled NaMN (Fig. 2*A*). To test this, unlabeled NMN was incubated with saturating levels of ^13^C_6_ nicotinic acid (M + 6) in the presence of recombinant CD38 enzyme and/or the small molecule CD38 inhibitor **78c** (51) (Fig. 2*B*). If base exchange were to occur, this would result in the formation of M + 6 labeled NaMN and unlabeled nicotinamide, though the formation of the latter product would also reflect the previously described NMN glycohydrolase activity of CD38 (49). This reaction was tested under both acidic (pH 4) and neutral (pH 7) conditions, as the base-exchange activity of CD38 toward NADP has been previously described to occur within the acidified environment of endolysosomes (41, 48), though the original identification of this base-exchange activity was found to occur at neutral pH (42, 45). In line with our hypothesis, incubation with this enzyme reduced NMN levels and resulted in the formation of M + 6 labeled NaMN (Fig. 2*B*) and unlabeled nicotinamide, although formation of the latter product also reflects its previously described NMN glycohydrolase activity. Unlike previously described base-exchange activity for CD38 that only occurred under acidic conditions (41, 52), we detected NMN base-exchange activity under both acidic (pH 4) and neutral (pH 7) conditions, which matches the original identification of NaADP synthesis through a base exchange reaction that occurred under neutral conditions (42).

One possibility could be that CD38 mediates a nonselective base exchange, swapping in either a nicotinic acid or a nicotinamide, in the latter case as a form of “autobase exchange” that would still yield NMN. To test whether this base-exchange reaction was selective, this previous reaction (Fig. 2*B*) was repeated with the addition of D_4_-nicotinamide (M + 4), which was present in equimolar amounts with ^13^C_6_ nicotinic acid (M + 6), along with NMN (Fig. 2*C*). The formation of M + 6 labeled NaMN was unaffected by the addition of D_4_-nicotinamide (M + 4), however M + 4 labeled NMN was also detected, albeit the formation of this product was not sensitive to the small molecule CD38 inhibitor **78c** (Fig. 2*C*). There was also a small amount of M + 4 labeled NaMN, and while it is conceivable that CD38 could first mediate the autobase exchange of NMN to yield M + 4 labeled NMN, followed by some previously undescribed base-exchange reaction, this product most likely reflects the detection of naturally occurring M + 1 isotopes of NMN. These isotopes would have the same molecular weight as NaMN, and this emphasizes the importance of our stable isotope labeling strategy in this investigation to better resolve the separation of these species. Finally, this reaction was also performed in the absence of base-exchange substrates, with NMN alone (Fig. 2*D*). As expected, in this condition there was no detection of M + 4 or M + 6 labeled NaMN (Fig. 2*D*), however the production of unlabeled nicotinamide due to CD38 still occurred at a similar level to when nicotinic acid was provided in the reaction (Fig. 2, *B* and *C*). This is in line with the previously described glycohydrolase activity and breakdown role of CD38 toward NMN (2, 49), suggesting that glycohydrolase activity occurs independently of, or could potentially precede, this base-exchange activity. The absolute magnitude of free nicotinamide formation, in comparison to labeled NaMN formation, would suggest that this glycohydrolase activity is more dominant than this newly described NMN base-exchange activity.

Next, we sought to further explore the selectivity of CD38-mediated base exchange between NMN and nicotinic acid by testing whether isomers of nicotinic acid would also act as base-exchange substrates with NMN (Fig. 2*E*). To test this, we generated 5,5-D_2_-ribose, D_4_-nicotinamide labeled NMN, containing an M + 2 label on the ribose group and an M + 4 label on the nicotinamide group for an overall mass shift of M + 6. This “double-labeled” NMN isotopolog should retain the M + 2 ribose label following loss or exchange of the M + 4 labeled nicotinamide group, which would result in a mass shift from M + 6 to M + 2. Double-labeled NMN (M + 6) was coincubated with unlabeled nicotinic acid, isonicotinic acid, and picolinic acid (Fig. 2*E*), which contain the carboxylic acid side group at meta, para, and ortho positions of the pyridine ring, respectively. Coincubation of double-labeled NMN (M + 6) with picolinic acid (pyridine-2-carboxylic acid) did not lead to the formation of M + 2 ribose labeled NaMN (Fig. 2*F*), in contrast to incubation with unlabeled nicotinic acid (pyridine-3-carboxylic acid) (Fig. 2*G*), which was in line with the previous experiment (Fig. 2, *B* and *C*). Interestingly, base exchange was also observed with isonicotinic acid (pyridine-4-carboxylic acid) (Fig. 2*H*). As in previous experiments (Fig. 2*C*), the formation of M + 2 labeled NaMN from both nicotinic acid and isonicotinic acid occurred at both pH 4 and pH 7 (Fig. 2, *G* and *H*). Representative chromatograms for the formation of this M + 2 labeled NaMN product are shown in Fig. 2*I*, with the major product eluting at around 17 min (Fig. 2*I*). For these latter two substrates, there was a small but detectable level of base exchange that occurred in the absence of CD38, indicating that this reaction can spontaneously occur in the absence of CD38. Interestingly, this spontaneous base exchange that occurred in the absence of CD38 was abolished by the presence of the small molecule inhibitor **78c** (51). This low level of spontaneous, nonenzymatic base exchange was not observed in the previous experiment (Fig. 2*C*), however this difference may reflect the impact of kinetic isotope effects (KIEs) from the incoming base, that is, ^13^C_6_-nicotinic acid or D_4_-nicotinamide, rather than unlabeled nicotinic acid isomers (Fig. 2*G*). These KIFs have been previously described for the nonenzymatic hydrolysis of NMN (53, 54, 55), and this difference might be especially pronounced where there is no enzymatic catalyst for exchange. The decrease in this low level of spontaneous base exchange is interesting and points to a potential interaction between the inhibitor **78c** and NMN. Previous modeling of the interaction between **78c** and CD38 suggests an interaction at its active site, with docking including a nearby interaction with ribose-5-phosphate. It is conceivable that a direct interaction between NMN and **78c** could impact the propensity of NMN for nonenzymatic base exchange.

A core insight that allowed for our discovery was the use of stable isotope labeling, as NMN and NaMN have similar spectral characteristics, similar elution times, and are almost identical in molecular weight, differing by only a single mass unit, with the signal from a lesser abundant NaMN potentially swamped by natural isotopes of NMN. One caveat to this approach is that heavy isotopes can impact enzyme function through KIEs. To ensure that this result was robust, and not an artefact of this particular isotope labelling combination, we next repeated this experiment using orthogonal labeling approaches, with three different combinations of isotope labels.

**Figure 3:**
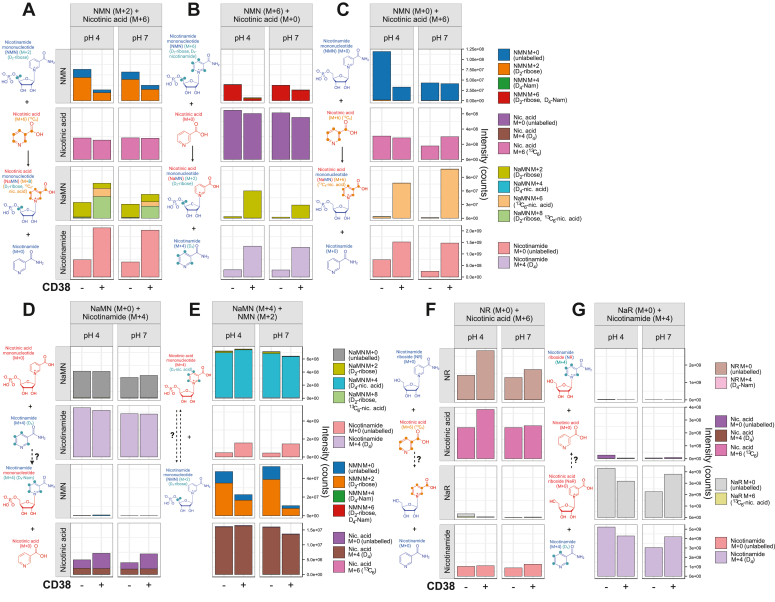
**CD38 base exchange is selective to NMN, and not NaMN, NR, or NaR**. CD38-mediated base exchange was confirmed through three different labelling combinations. *A*, 5,5-D_2_-ribose labelled NMN (M + 2) was coincubated with ^13^C_6_-nicotinic acid, leading to the formation of D_2_-ribose, ^13^C_6_-nicotinic acid labeled NaMN (M + 8). *B*, 5,5-D_2_-ribose, D_4_-nicotinamide–labeled NMN (M + 6) was coincubated with unlabeled nicotinic acid, leading to the formation of D_2_-ribose–labeled NaMN (M + 2). *C*, unlabeled NMN was coincubated with ^13^C_6_-nicotinic acid, leading to the formation of ^13^C_6_-nicotinic acid–labeled NaMN (M + 6). *D*, to test whether CD38 could mediate base exchange on NaMN rather than NMN, unlabeled NaMN was coincubated with D_4_-labeled nicotinamide (M + 4), however M + 4–labeled NMN was not identified. *E*, to test whether there is direct base exchange between NMN and NaMN, D_4_-nicotinic acid–labeled NaMN (M + 4) was coincubated with D_2_-ribose–labeled NMN (M + 2), however the hypothetical base exchange products were not detected. *F* and *G*, to test whether nicotinamide riboside (NR) or nicotinic acid riboside (NaR) could act as a substrate for base exchange, (*F*) unlabeled NR was coincubated with ^13^C_6_-nicotinic acid (M + 6), and (*G*) unlabeled NaR was coincubated with D_4_-nicotinamide (M + 4), with no base-exchange products observed in either case. Experiments were performed three separate times; data are shown from a single experiment that included all reaction combinations in parallel, using one technical observation per reaction condition. NaMN, nicotinic acid mononucleotide; NMN, nicotinamide mononucleotide.

This included generating 5,5-D_2_-ribose NMN, with an M + 2 label on the ribose group that should be retained following exchange of the nicotinamide group (Fig. 3*A*). This M + 2 ribose-labeled NMN was incubated with ^13^C_6_-nicotinic acid (M + 6) (Fig. 3*A*). In the presence of recombinant CD38, there was a reduction in overall NMN levels, with the production of M + 8–labeled NaMN, indicating that M + 2–labeled NMN had swapped its nicotinamide ring for a ^13^C_6_-nicotinic acid (M + 6), liberating a free, unlabeled nicotinamide group (Fig. 3*A*). This single-labeled NMN (M + 2) preparation was found to be impure, containing a minor degree of unlabeled NMN (M + 0), likely related to its enzymatic synthesis. As a result, there was also a lesser amount of M + 6–labeled NaMN in samples that were coincubated with CD38, reflecting base exchange of unlabeled NMN with ^13^C_6_-nicotinic acid (M + 6) (Fig. 3*A*). Both groups contained M + 2–labeled NaMN, however as described above, this likely reflects a signal from naturally occurring isotopes of NMN, as these two compounds differ by only single mass unit, with close elution times.

As in the previous experiment (Fig. 2*E*), we also used 5,5-D_2_-ribose, D_4_-nicotinamide–labeled NMN, containing an M + 2 label on the ribose group and an M + 4 label on the nicotinamide group for an overall mass shift of M + 6 (Fig. 3*B*). This double-labeled NMN (M + 6) was incubated with unlabeled nicotinic acid in the presence or absence of recombinant CD38. This led to the presence of M + 2–labeled NaMN, reflecting the loss of the M + 4–labeled nicotinamide group from NMN, and its replacement with unlabeled nicotinic acid (Fig. 3*B*). Consistent with this, this reaction liberated free M + 4–labeled nicotinamide, indicating its loss from M + 6 double-labeled NMN (Fig. 3*B*).

Finally, in line with our previous experiment (Fig. 2*A*), we again included the combination of unlabeled NMN with ^13^C_6_-nicotinic acid (M + 6), again yielding M + 6–labeled NaMN to indicate the exchange of the unlabeled nicotinamide ring of NMN with free ^13^C_6_-nicotinic acid (Fig. 3*C*). Unlike in Fig. 3*A*, where NaMN (M + 2) was observed due to additional natural isotope abundance from the original NMN (M + 2) label that would have differed by only a single mass unit, this isotope labeling used unlabeled NMN, which would have differed from NaMN (M + 2) by three mass units, rather than one. As a result, there was negligible detection of NaMN (M + 2) (Fig. 3*C*). Although in Fig. 3, *B* and *C* there might appear to be an impact of pH on NMN levels following CD38 treatment, this trend was not consistently observed across other, independently performed experiments. Despite this, we consistently observed that base-exchange activity, as measured by the formation of labeled NaMN, was not impacted by pH, which is in contrast to some descriptions of CD38 base-exchange activity in the acidic endolysosomes for its production of NaADP from NADP (41, 46, 47, 52). Together, these orthogonal approaches show that CD38 is capable of mediating base exchange on NMN with free nicotinic acid to yield NaMN, a deamidated intermediate of the Preiss–Handler/*de novo* pathways.

Having shown that CD38 could utilize nicotinic acid as a substrate for a base-exchange reaction (Figs. 2, *B* and *C*, 3, *B*–*C*), we next tested whether this reaction could occur in the reverse direction, that is, whether CD38 could carry out base exchange between NaMN and free nicotinamide to form NMN. Incubation of NaMN with D_4_-nicotinamide (M + 4) did not result in the formation of M + 4–labeled NMN (Fig. 3*D*), suggesting that this reaction only occurs in the direction of NMN to NaMN.

While unlikely, we also tested whether the nicotinic acid of NaMN could be used as a substrate for NMN base exchange through using NMN that was labeled at its ribose group only (M + 2), along with NaMN that was deuterated at the nicotinic acid group only (M + 4) (Fig. 3*E*). If base exchange was to occur here, this should result in the formation of M + 6–labeled NaMN. Unsurprisingly, this product was not observed (Fig. 3*E*). CD38 has strong glycohydrolase activity toward NMN (49), however in each of these experiments when CD38 was incubated with NaMN, we did not observe any reduction in its levels, in contrast to reductions in NMN, suggesting that NaMN is resistant to the glycohydrolase activity of this enzyme (Fig. 3*E*). This observation could have important implications for the use of NAD+ precursors as therapeutics, whereby NaMN, unlike the more commonly used NMN, is more resistant to hydrolysis by CD38.

In the experiment using unlabeled NaMN in Fig. 3*D*, we detected a small amount of nicotinic acid, most likely reflecting contamination or degradation of our original NaMN starting material: a similar level of baseline free nicotinamide was also present in other experiments involving NMN, rather than NaMN as a substrate. There was a slight trend toward increased nicotinic acid with CD38 treatment, however as described above, this was not correlated with decreased NaMN levels, suggesting that there is minimal NaMN cleavage by CD38. We similarly observed labeled nicotinic acid in Fig. 3*C*, also likely reflecting contamination of our NaMN isotope, which was in this case a custom synthesis using an enzyme-based protocol. For this reason, the baseline level of nicotinic acid contamination in this experiment is higher.

Finally, we also tested whether CD38 could mediate base exchange between NR and nicotinic acid, as has previously been described for another cell surface enzyme, CD157/BST1 (36). Unlike with NMN, when unlabeled NR was coincubated with ^13^C_6_-nicotinic acid (M + 6) in the presence of CD38, we did not observe M + 6–labeled NaR (Fig. 3*F*). Similarly, when unlabeled NaR was coincubated with D_4_-nicotinamide (M + 4), we could not detect the production of M + 4–labeled NR (Fig. 3*G*). Together, these data show that CD38-mediated base exchange between NMN and nicotinic acid to form NaMN is a new selective property of this enzyme, which does not carry out base exchange on NaMN, NR, or NaR (Fig. 3, *D*–*G*).

**Figure 4:**
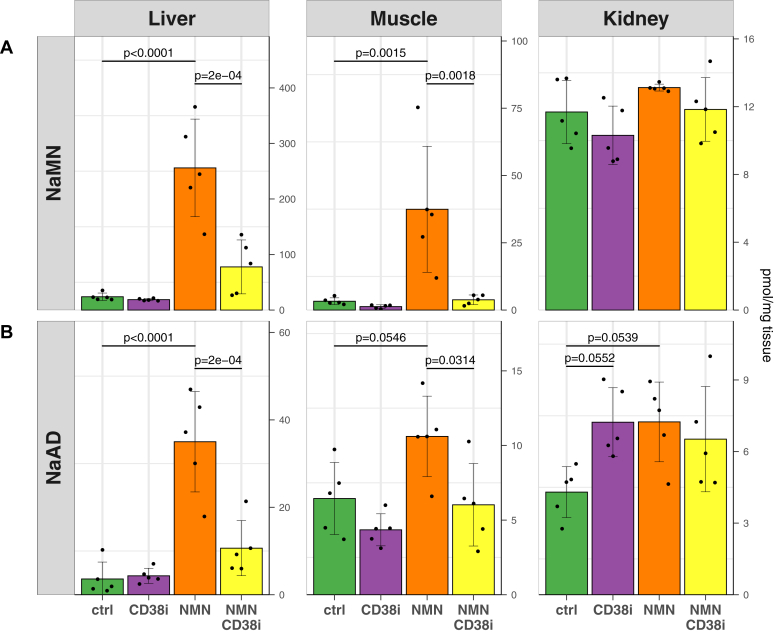
**CD38 inhibition blocks NMN-induced increases in NaMN and NaAD *in vivo***. Mice received a single oral dose of the amidated NAD^+^ precursor NMN (500 mg/kg) in the presence or absence of the small molecule CD38 inhibitor 78c (“CD38i”) (51) (10 mg/kg). Two hours later, animals were euthanized and liver, muscle (quadriceps), and kidneys were collected for metabolomics to measure levels of the Preiss–Handler/*de novo* pathway deamidated intermediates (*A*) nicotinic acid mononucleotide (NaMN) and (*B*) nicotinic acid adenine dinucleotide (NaAD). Each point represents data from a separate animal, data analyzed by two-way ANOVA followed by Tukey’s test for *post hoc* comparisons, n = 5/group, error bars are SD. NMN, nicotinamide mononucleotide.

Next, we sought to test whether the demonstration of *in vitro* NMN base-exchange activity (Figs. 2 and 3, *A*–*C*) was relevant to the formation of NaMN and NaAD following exogenous NMN treatment *in vivo*, as has been described in both humans and mice (23, 24). We used NMN treatment in C57BL6 mice in a 2∗2 experimental design, where animals received an acute oral bolus of NMN (500 mg/kg) or the CD38 small molecule inhibitor **78c** (51) (10 mg/kg) or both compounds together (Fig. 4). Two hours later, animals were euthanized, tissues rapidly dissected and preserved for metabolite extraction and metabolomics analysis. In line with previous work (23, 24), NMN treatment strongly increased levels of the deamidated metabolites NaMN and NaAD in liver and muscle, however this increase was abolished through coadministration of a CD38 inhibitor (Fig. 4). This reduction in NaMN and NaAD levels with **78c** treatment was not observed in kidney, however in this tissue, there was no NMN-induced spike in these metabolites to begin with: this may reflect the downstream bioavailability of orally delivered NMN to the kidney, including the likely metabolism and turnover of NMN by other tissues before it reaches the kidney. Alternatively, this could reflect reduced renal expression of CD38. Together, these findings (Fig. 4) helped to validate the physiological relevance of our *in vitro* discovery of the ability of CD38 to mediate the formation of NaMN from NMN through its base-exchange activity (Figs. 2 and 3, *A*–*C*). Further, they provide a compelling mechanistic explanation for the previously described increase in the deamidated metabolites NaMN and NaAD following exogenous administration with the amidated precursor NMN (23, 24).

## Discussion

The goal of this work was to identify additional mechanisms by which the amidated and deamidated arms of NAD^+^ biosynthesis could intersect in mammals. We showed that the cell surface enzyme CD38 can mediate the base exchange of the nicotinamide group of NMN for nicotinic acid, yielding NaMN. This discovery is consistent with work in the late 1980s from Tadayoshi Imai and colleagues, who described the existence of an NMN base-exchange reaction that was catalyzed by an unidentified protein isolated by column fractionation of spleen extracts (56, 57, 58). As with our findings (Fig. 3*D*), transglycosidation (base exchange) only occurred in the direction of NMN and nicotinic acid to NaMN, with no base exchange observed between NaMN and nicotinamide, suggesting that this reaction is irreversible (56). Similarly, base-exchange activity toward NR could not be detected, in line with our own results (Fig. 3*F*). Finally, NMN base-exchange activity from crude spleen extracts was also found to occur efficiently with isonicotinic acid as a substrate (58), as we also identified (Fig. 2*H*). Given its well-characterized expression in immune cells and our own findings here, we believe that CD38 is likely to be the enzyme responsible for the NMN base-exchange activity that was identified in this spleen extract (56, 57).

As with NMN, treatment with the amidated precursor NR in both mice and humans also leads to a sharp increase in the deamidated NAD^+^ precursor NaAD (21, 25). Similar to our own work, this was recently shown to occur *via* a base-exchange mechanism mediated by the cell surface enzyme CD157/BST1, whereby the nicotinamide group of NR was exchanged for a free nicotinic acid to yield NaR (36). This finding provided a strong mechanistic explanation for elevation of the deamidated arm of NAD biosynthesis following treatment with NR, however was exclusive to NR only, with no base exchange of CD157/BST1 observed toward NMN (36)–an activity that we now show can be mediated by a different enzyme, CD38 (Fig. 1*D*).

While not investigated here, it would be interesting to know whether CD38 also contributes to the impact of NR on NaAD levels, as in addition to its base exchange by CD157/BST1 into NaR, NR is also converted to NMN as an intermediate *en route* to NAD^+^ biosynthesis (17). CD38 is a membrane-bound protein; however its topology is not limited to facing the extracellular side of the plasma membrane: it can also protrude from the intracellular face of the plasma membrane and is also present on intracellular vesicle membranes such as endolysosomes (47, 59, 60). It would be interesting to know whether exogenous NR can then undergo base exchange by intracellular CD38 following its conversion to NMN. Of relevance to this question, it would be interesting to know whether the physiological role of CD38 in mediating the impact of NMN treatment on NaMN and NaAD levels (Fig. 4) occurs due to extracellular or intracellular facing CD38. NMN is barely detectable in serum, even after exogenous oral dosing, which has been previously explained by this being an intracellular metabolite, its measurements from circulation require analysis of whole blood, and correction for hematocrit. Given this, it is unclear whether extracellular facing CD38 plays a role in the results observed here. NMN enters the cell following its extracellular dephosphorylation into NR (61) and its incorporation into the NAD^+^ metabolome is blocked by deletion of NRK enzymes (62), with controversy around the existence of an intact NMN transporter (63, 64, 65). Assuming that NMN enters the cell in the form of NR and that CD38-mediated NMN base exchange is intracellular, it could be conceivable that CD38 might also play a role in the increase in NaMN and NaAD following NR treatment. This idea is however complicated by the evidence from Yaku *et al*., who show that the NR-induced spike in NaMN and NaAD is abolished in BST1 KO mice (36).

Another implication for the role of CD157/BST1 in mediating base exchange on NR (36) is not only to explain the formation of the deamidated intermediate NaAD but also in explaining the results of isotope labeling studies. Isotope tracing studies performed by Trammell *et al*. utilized double isotope–labeled NR, containing stable isotope labels at both the ribose and nicotinamide groups (25). When delivered *in vivo*, this double-labeled NR led to the formation of NaAD containing a single label only, along with predominantly single labeling of NAD^+^ (25). There are several possible explanations for the observation of single, rather than double labeling. First, once assimilated into NAD^+^, its rate of turnover (34) would lead to the separation of these labels, including the release of free nicotinamide that would be recycled back into NAD^+^ by the time tissues had been taken from animals following the initial NR bolus. Another possibility is that NR undergoes first pass metabolism in the gut and liver, being broken down into free nicotinamide prior to its cellular uptake and incorporation into the NAD^+^ metabolome (29, 34). This alone might explain single labeling of NAD^+^, but not the formation of single-labeled NaAD. Unlike mammals, bacteria in the gut microbiome express enzymes such as PncA, which can deamidate free nicotinamide into nicotinic acid, with the microbiome being responsible for the formation of nicotinic acid following administration with exogenous nicotinamide (30) and NR (29). If NR was being broken down into free nicotinamide, it is conceivable that its bacterial deamidation into nicotinic acid and assimilation into NAD^+^ through the Preiss–Handler pathway could account for the formation of single-labeled NaAD. Indeed, it has been proposed that NR is assimilated following its recycling between the host and gut microbiome *via* nicotinic acid as an intermediate (29). The discovery that CD157/BST1 also acts as an NR base-exchange enzyme (36) could also explain the formation of single labeled, deamidated intermediates (*i*.*e*. NaAD) following double-labeled NR treatment (25). It is, however, unclear as to what extent this base-exchange mechanism is physiologically relevant, compared to first pass metabolism of NR into free nicotinamide, and its subsequent bacterial deamidation into nicotinic acid (29, 34).

These questions related to NR metabolism are highly analogous to the findings presented here. As with NR, treatment with double-labeled NMN *in vivo* leads to relatively low levels of double-labeled NAD^+^ (24, 35). This could reflect the later time points used for those measurements, with intact NAD^+^ labeling likely to have undergone recycling (34) by that time point (24). It might also reflect its first pass metabolism in the gut and liver prior to uptake and incorporation (29, 34, 35). We had previously described that treatment with antibiotics to ablate the gut microbiome could shift the intact incorporation of double-labeled NMN into NAD^+^ to the salvage pathway, rather than the deamidated, NADS-dependent Preiss–Handler/*de novo* pathway (24). While that work demonstrated a role for the gut microbiome in deamidating NMN prior to its intact incorporation into NAD^+^, the CD38-dependent base-exchange mechanism described here could also account for the observation of single labeling of NAD^+^, as the nicotinamide group of NMN could be swapped out for an unlabeled nicotinic acid prior to its incorporation into NAD^+^.

This work also opens questions around other potential substrates for CD38-mediated base exchange. The reduced forms of NMN and NR, NMNH and NRH, respectively, were found to act as potent inducers of NAD levels, in some cases increasing NAD levels by more than 10-fold (22, 26, 28). The assimilation of NRH into the NAD^+^ metabolome differs from that of NR, being phosphorylated to NMNH *via* the enzyme adenosine kinase (22, 27), rather than NRK, as is the case for NR (17). As with NR and NMN, both NRH and NMNH treatment leads to sharp increase in levels of the deamidated metabolites NaMN and NaAD. It would be interesting to know whether CD38 has base-exchange activity toward those precursors, and for that matter, whether they are also susceptible to its glycohydrolase activity. This should be a goal for future work.

The degree to which base-exchange mechanisms contribute to the systemic metabolism of exogenous NR or NMN remains to be determined. While these data show a clear role for CD38 in the formation of the deamidated metabolites NaMN and NaAD following NMN treatment *in vivo* (Fig. 4), it is unclear to what degree flux through these intermediates contributes to overall NAD^+^ homeostasis. Future work should aim to address this, using time-resolved *in vivo* flux analysis of the NAD metabolism (6, 34) in the context of CD38 inhibition and stable isotope-labeled NMN, or in the context of CD157/BST1 deletion with labeled NR. It would be especially interesting to know in such an experiment what proportion of NaMN and NaAD contained an isotope label from an exogenous NMN bolus. Although adding another layer of difficulty to what would already be a demanding study, this work would be especially interesting if performed in the context of NADS KO mice (66), which could help to tease out the contributions of the deamidated pathway to the assimilation of NR and NMN into the NAD^+^ metabolome. Another strategy for teasing apart this contribution could be the use of CD38 KO mice, which could overcome uncertainty around the different levels of CD38 activity in different tissues, as well as potential complications from uneven tissue distribution and bioavailability of the inhibitor, and the likely uneven pharmacokinetics between the CD38 and NMN.

Another future experiment that would help to quantify the degree to which this base-exchange mechanism is relevant to the assimilation of exogenous NMN into NAD^+^ could be to use NMN that is labeled at the ribose and/or phosphate groups only and administer it in combination *in vivo* with labeled nicotinic acid in the presence or absence of a CD38 inhibitor. This could be an expensive experiment, due to the need for custom-labeled NMN and a saturating quantity of stable labeled nicotinic acid, however if successful, would provide a quantitative answer as to what proportion of exogenous NMN is incorporated following this base-exchange mechanism.

It is important to note that these results do not rule out the previously described (24, 29, 30) contributions of the gut microbiome to orally delivered NMN metabolism, and we consider it likely that a considerable proportion of orally delivered NMN undergoes breakdown in the gut to yield nicotinamide, which can be deamidated into nicotinic acid for cycling between the host and gut microbiome (29). It would be interesting in future work to conduct the above tracing experiments using either a CD38 inhibitor or CD38 KO mice or both, in the context of germ-free or antibiotics treated mice. Finally, given that our *in vitro* results suggest that this base-exchange reaction is specific to NMN, but not NaMN or NR (Fig. 3), it would be interesting to confirm this specificity *in vivo*, through parallel dosing with NR in the context of CD38 inhibition or deletion.

It is unclear whether this new NMN base-exchange activity of CD38 is more or less dominant than its previously described activity as an NMN glycohydrolase (49) which limits NMN levels *in vivo* (2). Our data (Fig. 2) show that CD38 mediates the formation of free nicotinamide from NMN regardless of the presence of nicotinic acid, suggesting that its glycohydrolase activity occurs prior to or independently of base-exchange activity. The quantitative amount of free nicotinamide formation exceeds that of NaMN formation, suggesting that glycohydrolase activity is more dominant, an idea supported by previous work establishing CD38 as a regulator for NMN availability *in vivo* (2). Arguing against this, in early work (56, 57) that described NMN transglycosylation activity from a spleen extract, NMN glycohydrolase activity (ν_H_) was also measured, and compared as a ratio against transglycosidase (base exchange, ν_T_) activity. In this work, transglycosidation activity was more dominant than glycohydrolase activity, with estimates of the ν_T_/ν_H_ ratio of between 2.3 (57) and 2.7 (56), however this could be increased by the addition of Triton X-100, and was competitively inhibited by free nicotinamide. Interestingly, later work using whole tissue lysates (58) demonstrated that overall NMN base-exchange activity exceeded glycohydrolase activity. This early work used a much higher concentration of substrates, with 8 mM NMN and 200 mM nicotinic acid, and demonstrated that the ratio between base-exchange and glycohydrolase activity (ν_T_/ν_H_ ratio) was strongly increased by higher substrate concentrations. In comparison, our study used much lower substrate concentrations, of only 0.2 mM NMN and 1 mM nicotinic acid.

In either case, this understanding still provides a mechanistic understanding for the sharp increase in the deamidated NAD^+^ precursors NaMN and NaAD, which could act as sensitive biomarkers (21, 25) for exogenous NAD^+^ precursor administration. Understanding whether this base-exchange pathway plays a physiologically relevant role for overall NAD^+^ homeostasis could, however, answer a more important question for the field. The rationale for using NMN and NR is that they bypass the rate-limiting step in NAD^+^ biosynthesis, which is the conversion of nicotinamide to NMN by the enzyme nicotinamide phosphoribosyltransferase (NAMPT) (67). If it is indeed the case that orally delivered NMN and NR undergo extensive first pass metabolism to yield free nicotinamide prior to its uptake and delivery to other tissues, one might expect that simply using free nicotinamide, as has long been used as nutrient fortification around the world, would be an equally effective strategy to raising NAD^+^ levels. Aside from having hypothetical bioequivalence, providing nicotinamide instead would also be much cheaper, given the costs of NR and NMN synthesis. Remarkably, these direct comparisons between different NAD^+^ precursors are largely lacking in animal models of disease (47). While there has been an explosion in studies that test the efficacy of NMN and NR in *in vivo* disease models, they are rarely conducted against a group treated with nicotinamide. One study (25) conducting a direct head-to-head comparison of NR against nicotinamide and nicotinic acid demonstrated differences in the pharmacokinetic profile of NAD^+^ formation, with NR treatment achieving a greater peak in NAD^+^ levels, along with differences in the formation of other NAD metabolites. One of the more convincing arguments that these precursors have superior efficacy as intact molecules is that NR treatment (68, 69) and NMN treatment (70, 71) can partially overcome the pathophysiological impacts of deletion of the rate-limiting enzyme NAMPT, which should not be possible if these precursors are degraded into free nicotinamide by first pass metabolism prior to their uptake (29, 34).

Here, we propose that CD38-mediated base exchange of NMN into NaMN could reconcile the low levels of intact NMN incorporation into NAD^+^ with its apparent efficacy as an NAD^+^ precursor that can bypass the deletion of the rate-limiting NAD biosynthetic enzyme NAMPT. Further, this mechanism could also explain the sharp increase in the deamidated metabolites NaMN and NaAD following NMN treatment. We propose that this is analogous to the base-exchange mechanism of NR, which is instead mediated by CD157/BST1 (36). Finally, this work expands our understanding of the role of CD38 in NAD^+^ homeostasis. This enzyme has received attention as a therapeutic target (51) due to its role as an NAD^+^ glycohydrolase (Fig. 1*B*) that controls overall NAD^+^ levels (1, 2, 3, 40), while also playing important signaling roles due to its base-exchange activity, which yields the signaling intermediate NaADP (41, 43, 48) (Fig. 1*C*). In this work, we show that this enzyme can also mediate base exchange on NMN to yield NaMN (Fig. 1*D*). Together, this provides a new mechanism by which the amidated and deamidated pathways of mammalian NAD biosynthesis can intersect.

There were several limitations to this study. We did not yet establish the precise enzyme kinetics of CD38-mediated base exchange on NMN, which will require detailed time-course experiments, including variations in substrate concentrations to test enzyme affinity. Notably, our experiments used supraphysiological concentrations of NAD^+^ substrates, with levels of NMN and nicotinic acid that are well above those found in tissues, and testing a broader range of substrate concentrations should be a core goal for the detailed characterization of enzyme kinetics of this reaction. While this has been well established for other activities of CD38, for example, its glycohydrolase activity toward NMN, these were able to utilize measurements based on changes in optical absorbance, allowing for higher throughput, plate reader–based time-resolved measurements (49). In our case, we were not able to show differences in absorbance of fluorescence characteristics between each of the substrates and products of our reactions, forcing us to rely on targeted mass spectrometry with stable isotope-labeled substrates. Resolution of each of these metabolites by mass spectrometry also relied on chromatographic separation, which together required around 30 min in mass spectrometry run time per individual sample, limiting our overall throughput due to cost and instrument time restraints. It is tempting to use the spectrophotometry assays that were described by Imai and colleagues (56, 57, 58, 72) for high-throughput measurements with a 96-well plate reader to characterize the enzyme kinetics of CD38. Unfortunately, those assays relied on high concentrations (2 mol/L) of cyanide as a conjugate, which altered the absorbance properties of NMN and NaMN. Due to local laboratory safety standards and its risk profile, use of this highly toxic reagent to conduct these assays was not possible. Regardless, future studies should aim to establish these parameters for the base exchange of NMN and nicotinic acid to NaMN, including Michaelis–Menten kinetics (including K_M_ and V_max_) and K_cat_. It would be especially interesting to determine whether this newly discovered activity is sensitive to the abundance of other NAD^+^ metabolites and whether the reaction can be limited by product inhibition. Further, as described in earlier sections, it would be interesting to quantify and compare the relative NMN glycohydrolase cleavage activity of CD38 compared to its activity an NMN base-exchange enzyme, including whether its base-exchange activity is secondary to its glycohydrolase activity. One analogy for this is the ability of CD38 to act as an NAD^+^ glycohydrolase, whereby most of the reaction products are nicotinamide and ADP ribose, however a small fraction of the product is converted to cyclic ADP ribose. It would also be interesting to compare the base-exchange activity of CD38 with the enzymes SARM1 and BST1, which also have base-exchange activity (36, 37). Another limitation to this study was that our *in vivo* studies used a small molecule CD38 inhibitor only—in future, further robust confirmation of these results would occur through the use of CD38 KO mice (44, 73). Future *in vivo* studies should also include measurement in other tissues, especially the small intestine, which has the highest level of CD38 expression (36). Another experimental aspect that could be optimized is the concentration of this inhibitor, which was in excess of the Ki of 78c for CD38, which is 8.4 nM (74). Finally, as described above, while we showed that CD38 played a key role in the formation of the deamidated metabolites NaMN and NaAD following *in vivo* administration with the amidated metabolite NMN, it would be interesting to determine the quantitative extent to which this base-exchange activity contributed to the overall incorporation of NMN into NAD^+^ *in vivo*.

## Methods

### Mass spectrometry

Metabolites were extracted from tissues using an extraction buffer containing 40:40:20 methanol, acetonitrile, and 0.1 N formic acid with the addition of 1 μM thymine-D_4_ as an internal standard. Briefly, snap-frozen tissue samples were crushed into a powder using a mortar and pestle with a small amount of liquid nitrogen sitting on a bed of dry ice. From this powder, 20 mg was weighed and 300 μl extraction buffer (previously cooled to −30 °C) was added. Samples were then lysed in a precooled Precellys Cryolys at 6500 rpm for 30 s, followed by a 20 s wait, and repeated twice more. Samples were then centrifuged for 10 min at 14,000*g* at 4 °C. Supernatants were collected and dried down in a vacuum centrifuge with no heat. Dried samples were reconstituted in 200 μl of a solution containing a 10:90 ratio of mobile phase A (20 mM ammonium acetate, pH 9) to mobile phase B (100% acetonitrile).

For data shown in Figs. 2 and 3, samples were run on a Thermo TSQ Vantage Triple-Stage Quadrupole mass spectrometry, coupled to a Vanquish HPLC system (Thermo) using an InfinityLab Poroshell 120 HILIC-Z column (2.1 × 150 mm, 2.7 μm, Agilent catalog number 673775-924). The injection volume was 5 μl, and the instrument was set to a dwell time of 30 ms, with a Q1 resolution of 0.7 and Q3 resolution of 1.2, with collision induced dissociation gas (mTorr) at 1.5, using selected reaction monitoring parameters shown in Table 1 below. The flow rate was 200 μl/min with the percentage of solvent B set at 90% (0–2 min), 70% (13 min), 40% (16–20 min), and 90% (21–30 min). Metabolites of interest eluted from this ingredient as follows: NR at 17.2 min, NaMN at 17.09 min, NMN at 17.3 min, NaR at 12.96 min, nicotinamide at 2.24 min, and nicotinic acid at 2.04 min.

**Table 1:** Selected reaction monitoring parameters for the targeted detection of metabolites shown in Figs. 2 and 3

Compound	Start time (min)	End time (min)	Polarity	Precursor (*m/z*)	Product (*m/z*)	Collision energy (V)	RF lens (V)
NA_80_0_0	0	30	Positive	124.038	80.071	22.47	78
NA_80_4_4	0	30	Positive	128.038	84.071	22.47	78
NA_80_4_3	0	30	Positive	128.038	83.071	22.47	78
NA_80_6_5	0	30	Positive	130.038	85.071	22.47	78
NaR_124_0_0	0	30	Positive	256.087	124.042	10.91	41
NaR_124_4_4	0	30	Positive	260.087	128.042	10.91	41
NaR_124_6_6	0	30	Positive	262.087	130.042	10.91	41
NaMN_124_0_0	0	30	Positive	336	123.929	14.28	53
NaMN_124_2_0	0	30	Positive	338	123.929	14.28	53
NaMN_124_2_2	0	30	Positive	338	125.929	14.28	53
NaMN_124_4_4	0	30	Positive	340	127.929	14.28	53
NaMN_124_4_2	0	30	Positive	340	125.929	14.28	53
NaMN_124_6_4	0	30	Positive	342	127.929	14.28	53
NaMN_124_6_6	0	30	Positive	342	129.929	14.28	53
NaMN_124_8_6	0	30	Positive	344	129.929	14.28	53
NAM_80_0_0	0	30	Positive	123.038	80.071	21.49	59
NAM_80_4_4	0	30	Positive	127.038	84.071	21.49	59
NAM_80_4_3	0	30	Positive	127.038	83.071	21.49	59
NR_123_0_0	0	30	Positive	255	123	23.5	64
NR_123_4_4	0	30	Positive	259	127	23.5	64
NMN_123_0_0	0	30	Positive	334.962	123	14.89	138
NMN_123_2_0	0	30	Positive	336.962	123	14.89	138
NMN_123_4_4	0	30	Positive	338.962	127	14.89	138
NMN_123_6_4	0	30	Positive	340.962	127	14.89	138

For data shown in Fig. 4, samples were run on a 1260 Infinity LC system using an Amide XBridge BEH column (100 × 2.1 mm, Waters Corp) coupled to a QTRAP 500 mass spec (SCIEX), as described previously (24).

### Enzyme assays

CD38 base-exchange activity was assessed *in vitro* using recombinant human CD38 (UniProt P28907, Val54–Ile300) corresponding to its extracellular domain, with a C-terminal 6xHis tag produced in NS0-derived mouse myeloma cell line from R&D Systems (catalog number RDS2404-AC), with activity of >2500 pmol/min/μg, as measured by its ability to convert nicotinamide guanine dinucleotide to cyclic GDP ribose. The CD38 small molecule inhibitor used here was 4-[[*trans*-4-(2-methoxyethoxy)cyclohexyl]amino]-1-methyl-6-(5-thiazolyl)-2(1*H*)-quinolinone (CAS #1700637-55-3), as described as compound **78c** in Haffner *et al*. (51). Reactions were carried out in 30 mM Tris, which was adjusted to pH 4 or pH 7 as shown in each figure. NMN and NaMN isotopologs were obtained under custom synthesis from GeneHarbor Biotechnology (Hong Kong Science and Technology Park), who utilize an enzymatic method for synthesis. We obtained ^13^C_6_-nicotinic acid (CLM-9954) and D_4_-nicotinamide (DLM-6883) from Cambridge Isotope Laboratories. Base exchange was assayed with a 1:5 ratio of base-exchange substrate (*e*.*g*. NMN, NaMN, NR, NaR) to free base (*e*.*g*. nicotinic acid, nicotinamide). Reactions took place in 6 μl volumes, containing 250 mM sucrose, 10 μg/ml bovine serum albumin, 30 mM Tris buffer that was adjusted to either pH 4 or pH 7, with 0.2 mM base-exchange substrate (*e*.*g*. NMN, NaMN, NR, NaR) and 1 mM free base (nicotinic acid or nicotinamide), with or without the CD38 inhibitor 78c at 20 μM, with or without recombinant CD38 (3 ng per 10 μl reaction). Reactions were allowed to proceed for 30 min at room temperature and quenched through the addition of 50 ul of a 1:1 mix of mobile phase A (20 mM ammonium acetate, pH 9) and mobile phase B (acetonitrile). Reaction products were then dried down under vacuum centrifuge for 2 h, prior to resuspension for mass spectrometry.

### Animal studies

All experiments were approved by the UNSW Animal Care and Ethics Committee, which operates under the animal ethics guidelines from the National Health and Medical Research Council of Australia. Animals had ad libitum access to standard chow diet (Gordon’s stock feeds) and acidified drinking water, and were maintained on a 12 h light/dark cycle at 22 °C and 80% humidity, in individually ventilated cages. Male C57BL/6J mice were obtained from the Animal Resource Centre in Perth, and allowed to acclimatize for at least 1 week prior to experiments. At 9 weeks of age, animals received a single dose of NMN (500 mg/kg) and/or the CD38 inhibitor 78c (10 mg/kg), for a 2∗2 study design. Both compounds were delivered by oral gavage, and 2 h later animals were euthanized and tissues rapidly dissected and snap-frozen for mass spectrometry.

To prepare the CD38 inhibitor **78c** for *in vivo* administration, we first prepared a solution of 4 mg/ml 78c in 20% Capsitol in milliQ water. This was adjusted to pH 3 using citric acid powder, heated to 80 °C on a hot plate, with the addition of 5% Solutol (Kalliphor HS15), and stirred for 15 min. Following this, the solution was left in a sonicating water bath for 30 min, prior to its use on the same day. This was then delivered to mice by oral gavage at a dose of 10 mg/kg. NMN was prepared for oral gavage through freshly diluting dry NMN powder (GeneHarbor Biotechnology) in PBS prior to its oral gavage at a dose of 500 mg/kg.

### Statistics

Data (Fig. 4) were analyzed by two-way ANOVA followed by Tukey’s honest significant difference *post hoc* test for between treatment comparisons. Error bars in figures indicate SDs. Data were analyzed in *R* and figures generated using the R packages *ggplot2*, *ggh4x*, and *rstatix*. A detailed, annotated R script showing our analysis has been uploaded to our open data site. Please see “data availability” section below.

## Data availability

Mass spectrometry RAW files, CSV files, and annotated R scripts and data files allowing for reproducible data analysis and the recreation of figures presented here have been uploaded to the Mendeley Data server, reserved https://doi.org/10.17632/k72zvgyst6.1 (preview link).

## Conflicts of interest

L. E. W. is a scientific advisor to Metro International Biotech, which is developing NAD^+^ precursors and their derivatives as therapies for age-related disease, and is a shareholder in EdenRoc Sciences, the parent company of Metro Biotech. He is also a cofounder, advisor, and shareholder in Jumpstart Fertility Inc, which is targeting the NAD^+^ metabolome to improve embryo culture media in assisted reproduction. The other authors declare that they have no conflicts of interest with the contents of this article.

## References

[1] Camacho-Pereira J., Tarrago M.G., Chini C.C.S., Nin V., Escande C., Warner G.M. (2016). CD38 dictates age-related NAD decline and mitochondrial dysfunction through an SIRT3-dependent mechanism. Cell Metab..

[2] Chini C.C.S., Peclat T.R., Warner G.M., Kashyap S., Espindola-Netto J.M., de Oliveira G.C. (2020). CD38 ecto-enzyme in immune cells is induced during aging and regulates NAD(+) and NMN levels. Nat. Metab..

[3] Covarrubias A.J., Kale A., Perrone R., Lopez-Dominguez J.A., Pisco A.O., Kasler H.G. (2020). Senescent cells promote tissue NAD(+) decline during ageing via the activation of CD38(+) macrophages. Nat. Metab..

[4] Fang E.F., Kassahun H., Croteau D.L., Scheibye-Knudsen M., Marosi K., Lu H. (2016). NAD(+) replenishment improves lifespan and healthspan in ataxia telangiectasia models via mitophagy and DNA repair. Cell Metab.

[5] McReynolds M.R., Chellappa K., Baur J.A. (2020). Age-related NAD(+) decline. Exp. Gerontol..

[6] McReynolds M.R., Chellappa K., Chiles E., Jankowski C., Shen Y., Chen L. (2021). NAD(+) flux is maintained in aged mice despite lower tissue concentrations. Cell Syst.

[7] Aflatounian A., Paris V.R., Richani D., Edwards M.C., Cochran B.J., Ledger W.L. (2022). Declining muscle NAD(+) in a hyperandrogenism PCOS mouse model: possible role in metabolic dysregulation. Mol. Metab..

[8] Bertoldo M.J., Listijono D.R., Ho W.J., Riepsamen A.H., Goss D.M., Richani D. (2020). NAD(+) repletion female fertility during reproductive aging. Cell Rep.

[9] Das A., Huang G.X., Bonkowski M.S., Longchamp A., Li C., Schultz M.B. (2018). Impairment of an endothelial NAD(+)-H2S signaling network is a reversible cause of vascular aging. Cell.

[10] Habibalahi A., Campbell J.M., Bertoldo M.J., Mahbub S.B., Goss D.M., Ledger W.L. (2022). Unique deep radiomic signature shows NMN treatment reverses morphology of oocytes from aged mice. Biomedicines.

[11] Ho W.J., Marinova M.B., Listijono D.R., Bertoldo M.J., Richani D., Kim L.J. (2024). Fertility protection during chemotherapy treatment by boosting the NAD(P)(+) metabolome. EMBO Mol Med..

[12] Mouchiroud L., Houtkooper R.H., Moullan N., Katsyuba E., Ryu D., Canto C. (2013). The NAD(+)/Sirtuin pathway modulates longevity through activation of mitochondrial UPR and FOXO signaling. Cell.

[13] Wu L.E., Sinclair D.A. (2016). Restoring stem cells - all you need is NAD(.). Cell Res.

[14] Zhang H., Ryu D., Wu Y., Gariani K., Wang X., Luan P. (2016). NAD(+) repletion improves mitochondrial and stem cell function and enhances life span in mice. Science.

[15] Mills K.F., Yoshida S., Stein L.R., Grozio A., Kubota S., Sasaki Y. (2016). Long-term administration of nicotinamide mononucleotide mitigates age-associated physiological decline in mice. Cell Metab.

[16] Belenky P., Christensen K.C., Gazzaniga F., Pletnev A.A., Brenner C. (2009). Nicotinamide riboside and nicotinic acid riboside salvage in fungi and mammals. Quantitative basis for Urh1 and purine nucleoside phosphorylase function in NAD+ metabolism. J. Biol. Chem..

[17] Bieganowski P., Brenner C. (2004). Discoveries of nicotinamide riboside as a nutrient and conserved NRK genes establish a Preiss-Handler independent route to NAD+ in fungi and humans. Cell.

[18] Tempel W., Rabeh W.M., Bogan K.L., Belenky P., Wojcik M., Seidle H.F. (2007). Nicotinamide riboside kinase structures reveal new pathways to NAD+. Plos Biol..

[19] Preiss J., Handler P. (1958). Biosynthesis of diphosphopyridine nucleotide. I. Identification of intermediates. J. Biol. Chem..

[20] Preiss J., Handler P. (1958). Biosynthesis of diphosphopyridine nucleotide. II. Enzymatic aspects. J. Biol. Chem..

[21] Elhassan Y.S., Kluckova K., Fletcher R.S., Schmidt M.S., Garten A., Doig C.L. (2019). Nicotinamide riboside augments the aged human skeletal muscle NAD(+) metabolome and induces transcriptomic and anti-inflammatory signatures. Cell Rep.

[22] Giroud-Gerbetant J., Joffraud M., Giner M.P., Cercillieux A., Bartova S., Makarov M.V. (2019). A reduced form of nicotinamide riboside defines a new path for NAD(+) biosynthesis and acts as an orally bioavailable NAD(+) precursor. Mol. Metab..

[23] Igarashi M., Nakagawa-Nagahama Y., Miura M., Kashiwabara K., Yaku K., Sawada M. (2022). Chronic nicotinamide mononucleotide supplementation elevates blood nicotinamide adenine dinucleotide levels and alters muscle function in healthy older men. NPJ Aging.

[24] Kim L.J., Chalmers T.J., Madawala R., Smith G.C., Li C., Das A. (2023). Host-microbiome interactions in nicotinamide mononucleotide (NMN) deamidation. FEBS Lett..

[25] Trammell S.A., Schmidt M.S., Weidemann B.J., Redpath P., Jaksch F., Dellinger R.W. (2016). Nicotinamide riboside is uniquely and orally bioavailable in mice and humans. Nat. Commun..

[26] Yang Y., Mohammed F.S., Zhang N., Sauve A.A. (2019). Dihydronicotinamide riboside is a potent NAD(+) concentration enhancer in vitro and in vivo. J. Biol. Chem..

[27] Yang Y., Zhang N., Zhang G., Sauve A.A. (2020). NRH salvage and conversion to NAD(+) requires NRH kinase activity by adenosine kinase. Nat. Metab..

[28] Zapata-Perez R., Tammaro A., Schomakers B.V., Scantlebery A.M.L., Denis S., Elfrink H.L. (2021). Reduced nicotinamide mononucleotide is a new and potent NAD(+) precursor in mammalian cells and mice. FASEB J..

[29] Chellappa K., McReynolds M.R., Lu W., Zeng X., Makarov M., Hayat F. (2022). NAD precursors cycle between host tissues and the gut microbiome. Cell Metab..

[30] Shats I., Williams J.G., Liu J., Makarov M.V., Wu X., Lih F.B. (2020). Bacteria boost mammalian host NAD metabolism by engaging the deamidated biosynthesis pathway. Cell Metab.

[31] Galeazzi L., Bocci P., Amici A., Brunetti L., Ruggieri S., Romine M. (2011). Identification of nicotinamide mononucleotide deamidase of the bacterial pyridine nucleotide cycle reveals a novel broadly conserved amidohydrolase family. J. Biol. Chem..

[32] Petrack B., Greengard P., Craston A., Sheppy F. (1965). Nicotinamide deamidase from mammalian liver. J. Biol. Chem..

[33] Sarma D.S., Rajalakshmi S., Sarma P.S. (1961). Deamidation of nicotinamide and NMN. Biochem. Biophys. Res. Commun..

[34] Liu L., Su X., Quinn W.J., Hui S., Krukenberg K., Frederick D.W. (2018). Quantitative analysis of NAD synthesis-breakdown Fluxes. Cell Metab..

[35] Sauve A.A., Wang Q., Zhang N., Kang S., Rathmann A., Yang Y. (2023). Triple-isotope tracing for pathway discernment of NMN-induced NAD(+) biosynthesis in whole mice. Int. J. Mol. Sci..

[36] Yaku K., Palikhe S., Izumi H., Yoshida T., Hikosaka K., Hayat F. (2021). BST1 regulates nicotinamide riboside metabolism via its glycohydrolase and base-exchange activities. Nat. Commun..

[37] Angeletti C., Amici A., Gilley J., Loreto A., Trapanotto A.G., Antoniou C. (2022). SARM1 is a multi-functional NAD(P)ase with prominent base exchange activity, all regulated bymultiple physiologically relevant NAD metabolites. iScience.

[38] Peclat T.R., Agorrody G., Colman L., Kashyap S., Zeidler J.D., Chini C.C.S. (2024). Ecto-CD38-NADase inhibition modulates cardiac metabolism and protects mice against doxorubicin-induced cardiotoxicity. Cardiovasc. Res..

[39] Perrone R., Ashok Kumaar P.V., Haky L., Hahn C., Riley R., Balough J. (2023). CD38 regulates ovarian function and fecundity via NAD(+) metabolism. iScience.

[40] Tarrago M.G., Chini C.C.S., Kanamori K.S., Warner G.M., Caride A., de Oliveira G.C. (2018). A potent and specific CD38 inhibitor ameliorates age-related metabolic dysfunction by reversing tissue NAD(+) decline. Cell Metab.

[41] Aarhus R., Graeff R.M., Dickey D.M., Walseth T.F., Lee H.C. (1995). ADP-ribosyl cyclase and CD38 catalyze the synthesis of a calcium-mobilizing metabolite from NADP. J. Biol. Chem..

[42] Bernofsky C. (1980). Nicotinic acid adenine dinucleotide phosphate (NAADP+). Methods Enzymol..

[43] Chini E.N., Beers K.W., Dousa T.P. (1995). Nicotinate adenine dinucleotide phosphate (NAADP) triggers a specific calcium release system in sea urchin eggs. J. Biol. Chem..

[44] Chini E.N., Chini C.C., Kato I., Takasawa S., Okamoto H. (2002). CD38 is the major enzyme responsible for synthesis of nicotinic acid-adenine dinucleotide phosphate in mammalian tissues. Biochem. J..

[45] Chini E.N., Dousa T.P. (1995). Enzymatic synthesis and degradation of nicotinate adenine dinucleotide phosphate (NAADP), a Ca(2+)-releasing agonist, in rat tissues. Biochem. Biophys. Res. Commun..

[46] Fang C., Li T., Li Y., Xu G.J., Deng Q.W., Chen Y.J. (2018). CD38 produces nicotinic acid adenosine dinucleotide phosphate in the lysosome. J. Biol. Chem..

[47] Li C., Wu L.E. (2021). Risks and rewards of targeting NAD(+) homeostasis in the brain. Mech. Ageing Dev..

[48] Nam T.S., Park D.R., Rah S.Y., Woo T.G., Chung H.T., Brenner C. (2020). Interleukin-8 drives CD38 to form NAADP from NADP(+) and NAAD in the endolysosomes to mobilize Ca(2+) and effect cell migration. FASEB J..

[49] Sauve A.A., Munshi C., Lee H.C., Schramm V.L. (1998). The reaction mechanism for CD38. A single intermediate is responsible for cyclization, hydrolysis, and base-exchange chemistries. Biochemistry.

[50] Preugschat F., Tomberlin G.H., Porter D.J. (2008). The base exchange reaction of NAD+ glycohydrolase: identification of novel heterocyclic alternative substrates. Arch. Biochem. Biophys..

[51] Haffner C.D., Becherer J.D., Boros E.E., Cadilla R., Carpenter T., Cowan D. (2015). Discovery, synthesis, and biological evaluation of thiazoloquin(az)olin(on)es as potent CD38 inhibitors. J. Med. Chem..

[52] Graeff R.M., Franco L., De Flora A., Lee H.C. (1998). Cyclic GMP-dependent and -independent effects on the synthesis of the calcium messengers cyclic ADP-ribose and nicotinic acid adenine dinucleotide phosphate. J. Biol. Chem..

[53] Bull H.G., Ferraz J.P., Cordes E.H., Ribbi A., Apitz-Castro R. (1978). Concerning the mechanism of the enzymatic and nonenzymatic hydrolysis of nicotinamide nucleotide coenzymes. J. Biol. Chem..

[54] Skoog M.T. (1986). Mechanism and activation for allosteric adenosine 5'-monophosphate nucleosidase. Kinetic alpha-deuterium isotope effects for the enzyme-catalyzed hydrolysis of adenosine 5'-monophosphate and nicotinamide mononucleotide. J. Biol. Chem..

[55] Tarnus C., Schuber F. (1987). Application of linear free-energy relationships to the mechanistic probing of nonenzymatic and NAD+-glycohydrolase-catalyzed hydrolysis of pyridine dinucleotides. Bioorg. Chem..

[56] Imai T. (1989). Kinetic analysis of the transglycosidation reaction catalyzed by rabbit spleen pyridine nucleotide glycohydrolase. J. Biochem..

[57] Imai T. (1989). Purification and characterization of a pyridine nucleotide glycohydrolase from rabbit spleen. J. Biochem..

[58] Imai T. (2002). Substrate specificity of mammalian pyridine nucleotide transglycosidases. J. Nutr. Sci. Vitaminol (Tokyo).

[59] Aksoy P., White T.A., Thompson M., Chini E.N. (2006). Regulation of intracellular levels of NAD: a novel role for CD38. Biochem. Biophys. Res. Commun..

[60] Zhao Y.J., Lam C.M., Lee H.C. (2012). The membrane-bound enzyme CD38 exists in two opposing orientations. Sci. Signal.

[61] Ratajczak J., Joffraud M., Trammell S.A., Ras R., Canela N., Boutant M. (2016). NRK1 controls nicotinamide mononucleotide and nicotinamide riboside metabolism in mammalian cells. Nat. Commun..

[62] Fletcher R.S., Ratajczak J., Doig C.L., Oakey L.A., Callingham R., Da Silva Xavier G. (2017). Nicotinamide riboside kinases display redundancy in mediating nicotinamide mononucleotide and nicotinamide riboside metabolism in skeletal muscle cells. Mol. Metab..

[63] Grozio A., Mills K.F., Yoshino J., Bruzzone S., Sociali G., Tokizane K. (2019). Slc12a8 is a nicotinamide mononucleotide transporter. Nat. Metab..

[64] Schmidt M.S., Brenner C. (2019). Absence of evidence that Slc12a8 encodes a nicotinamide mononucleotide transporter. Nat. Metab..

[65] Wu L.E., Sinclair D.A. (2019). The elusive NMN transporter is found. Nat. Metab..

[66] Szot J.O., Cuny H., Martin E.M., Sheng D.Z., Iyer K., Portelli S. (2024). A metabolic signature for NADSYN1-dependent congenital NAD deficiency disorder. J. Clin. Invest.

[67] Revollo J.R., Grimm A.A., Imai S. (2004). The NAD biosynthesis pathway mediated by nicotinamide phosphoribosyltransferase regulates Sir2 activity in mammalian cells. J. Biol. Chem..

[68] Frederick D.W., Loro E., Liu L., Davila A., Chellappa K., Silverman I.M. (2016). Loss of NAD homeostasis leads to progressive and reversible degeneration of skeletal muscle. Cell Metab.

[69] Mukherjee S., Chellappa K., Moffitt A., Ndungu J., Dellinger R.W., Davis J.G. (2017). Nicotinamide adenine dinucleotide biosynthesis promotes liver regeneration. Hepatology.

[70] Stromsdorfer K.L., Yamaguchi S., Yoon M.J., Moseley A.C., Franczyk M.P., Kelly S.C. (2016). NAMPT-mediated NAD(+) biosynthesis in adipocytes regulates adipose tissue function and multi-organ insulin sensitivity in mice. Cell Rep.

[71] Wang X., Zhang Q., Bao R., Zhang N., Wang Y., Polo-Parada L. (2017). Deletion of nampt in projection neurons of adult mice leads to motor dysfunction, neurodegeneration, and death. Cell Rep.

[72] Imai T. (1979). Isolation and properties of a glycohydrolase specific for nicotinamide mononucleotide from Azotobacter vinelandii. J. Biochem..

[73] Kato I., Yamamoto Y., Fujimura M., Noguchi N., Takasawa S., Okamoto H. (1999). CD38 disruption impairs glucose-induced increases in cyclic ADP-ribose, [Ca2+]i, and insulin secretion. J. Biol. Chem..

[74] Boslett J., Reddy N., Alzarie Y.A., Zweier J.L. (2019). Inhibition of CD38 with the Thiazoloquin(az)olin(on)e 78c Protects the Heart against Postischemic Injury. J. Pharmacol. Exp. Ther..

